# Iodine status and supplementation in pregnancy: an overview of the evidence provided by meta-analyses

**DOI:** 10.1007/s11154-022-09760-7

**Published:** 2022-10-13

**Authors:** Laura Croce, Luca Chiovato, Massimo Tonacchera, Elena Petrosino, Maria Laura Tanda, Mariacarla Moleti, Flavia Magri, Antonella Olivieri, Elizabeth N. Pearce, Mario Rotondi

**Affiliations:** 1 Department of Internal Medicine and Therapeutics, University of Pavia, Pavia (PV), 27100 Pavia, Italy; 2 Istituti Clinici Scientifici Maugeri IRCCS, Unit of Internal Medicine and Endocrinology, Laboratory for Endocrine Disruptors, 27100 Pavia, PV Italy; 3 Endocrinology Section, Department of Clinical and Experimental Medicine, University of Pisa, Pisa, Italy; 4 Department of Medicine and Surgery, University of Insubria, Varese, Italy; 5 Department of Clinical and Experimental Medicine, University of Messina, Messina, Italy; 6 Department of Cardiovascular and Endocrine-Metabolic Diseases and Aging, Italian National Institute of Health, Rome, Italy; 7 Section of Endocrinology Diabetes, and Nutrition, Boston University School of Medicine, Boston, MA USA

**Keywords:** Iodine, Thyroid, Pregnancy, Hypothyroidism, Autoimmunity, Meta-analyses

## Abstract

Iodine supplementation during pregnancy in areas with mild-moderate deficiency is still a matter of debate. The present study aimed at systematically reviewing currently available evidences provided by meta-analyses with the aim to further clarify controversial aspects regarding the need of iodine supplementation in pregnancy as well as to provide guidance on clinical decision-making, even in areas with mild-moderate deficiency. Medline, Embase and Cochrane search from 1969 to 2022 were performed. For the purpose of this review, only studies containing meta-analytic data were selected. A total of 7 meta-analyses were retrieved. Four meta-analyses evaluated the relationship between iodine status during pregnancy and neonatal and maternal outcomes suggesting the existence of a U-shaped correlation between iodine status and several maternal and neonatal consequences, especially if iodine status is evaluated at the beginning of pregnancy. Three meta-analyses evaluating the results of intervention trials failed to provide straightforward conclusions on the benefits of iodine supplementation in pregnant women in areas with mild-moderate iodine deficiency. Although evidence coming from meta-analyses suggests a role of iodine status during pregnancy in determining maternal and child outcomes, results of meta-analyses of intervention trials are still controversial. Several factors including, degree of iodine deficiency, and pooling studies conducted in areas with different iodine intake, may account for the lack of benefits reported by meta-analyses of intervention trials. More high-quality, randomized, controlled trials including information on timing, dose and regimen of iodine supplementation are needed to further elucidate this issue.

## Introduction

Iodine is an essential trace element required for the synthesis of thyroid hormones [1, 2]. During pregnancy, a greater iodine intake is needed due to an increase in maternal thyroid hormone synthesis, iodine transfer to the fetus, and greater urinary iodine loss due to an increased glomerular filtration rate [3]. Severe iodine deficiency and, consequently, inadequate iodine availability for the fetus during pregnancy cause impaired thyroid hormone synthesis, resulting in an increased risk of maternal goiter, fetal goiter, growth retardation and brain damage [4–6].

Even in countries where effective salt iodization programs are established, several studies indicate that pregnant and lactating women may be at high risk of having inadequate iodine levels [7, 8]. Since iodized salt may not be a sufficient source of iodine to meet the minimum requirements of this vulnerable group, several international authorities recommend a daily supplement of 150 µg of iodine for pregnant and lactating women. While in countries with severe iodine deficiency (defined according to WHO, by a median Urinary Iodine Concentration in school-aged children lower than 20 µg/L[9]) the implementation of iodine supplementation clearly improves clinical outcomes [10], studies in areas of moderate or mild deficiency (with a median UIC between 20 and 49 µg/L and between 50 and 99 µg/L, respectively[9]), led to inconclusive results. Overall, the consequences of mild-to-moderate iodine deficiency during pregnancy on fetal development are less clear, since there is a great variability in the results of available studies. While some studies suggest that mild to moderate iodine deficiency could impair child neurodevelopment [11–16], other investigations failed to demonstrate negative effects [17, 18]. Similar discrepancies emerge when evaluating intervention trials [19]. Differences in results between trials may be due to methodological differences (e.g., measurement of iodine status, selected reference group, and available data on confounders), the different outcomes taken into account, the age at assessment of the neurodevelopmental outcome of interest, the timing of the iodine measurements, and the relative severity of iodine deficiency in the population.

Moreover, recent evidence suggests that excessive iodine intake could be detrimental both for maternal thyroid status or for pregnancy outcomes [18, 20–22].

Due to the relevant number of retrospective cohort studies and clinical trials on the effects of iodine supplementation in pregnancy, several meta-analyses attempted to clarify the current state of art, but questions remain unanswered. The present study collected and systematically reviewed currently available data from meta-analyses with the aim to clarify controversial aspects regarding the multifaceted role of iodine in pregnancy as well as to provide guidance on clinical decision-making.

## Materials and methods

**Fig. 1:**
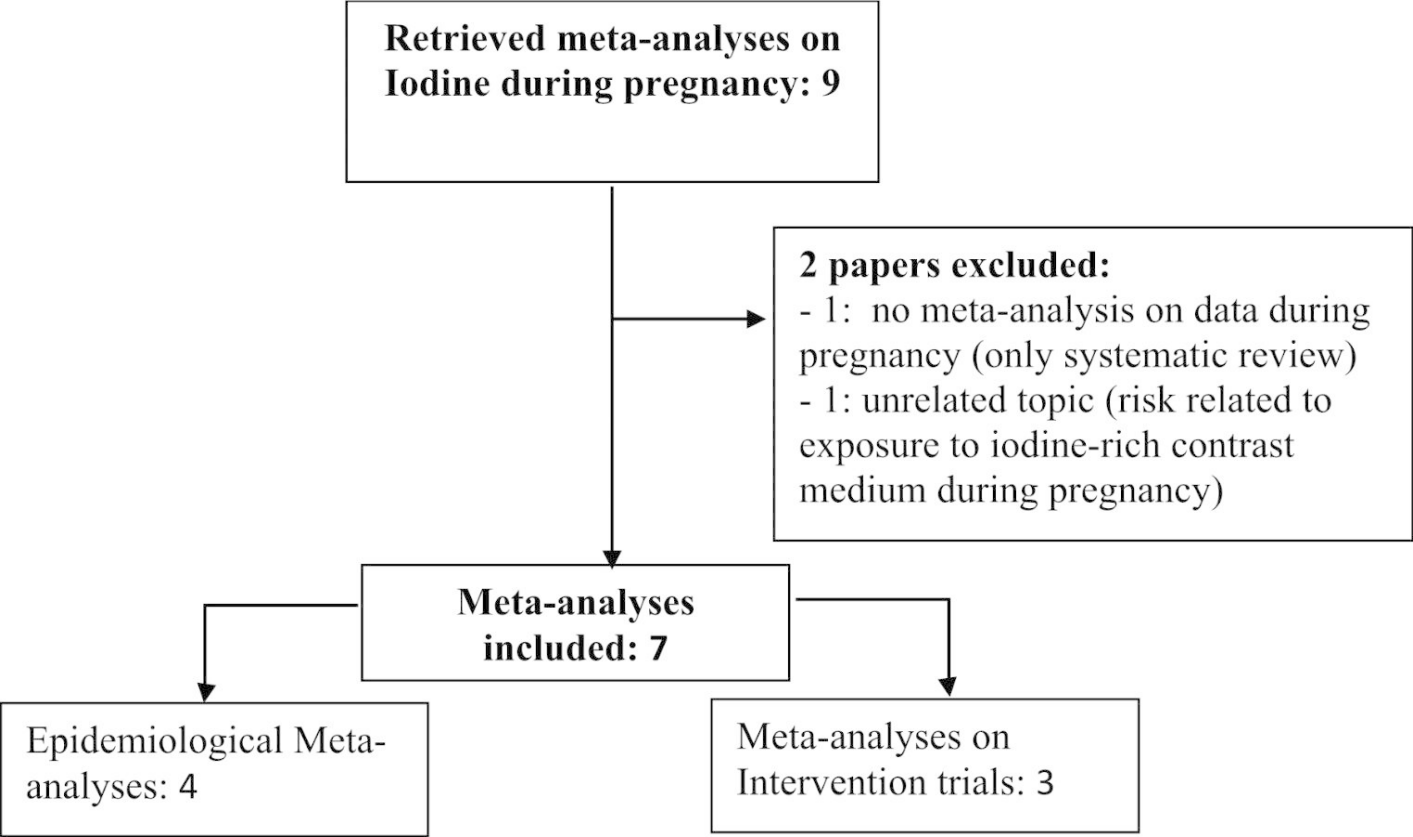
flow-chart summarizing the systematic review research process

A comprehensive narrative review was performed. We searched for relevant literature using Medline, Embase and Cochrane search and including the following terms: (“iodine“[MeSH Terms] OR (“iodine“[All Fields]) AND (“pregnancy” [MeSH Terms] OR “pregnancy”[All fields]) AND (“Meta-analysis” [MeSH Terms] OR “Meta-analysis” [All fields]). Publications from 1969 up to 2022 were included. For the purpose of this review, only studies containing meta-analytic data were selected. Nine meta-analyses meeting the required selection criteria were found [23–31]. One study [31] was excluded due to the absence of meta-analytic data on pregnancy, and another one [30] was excluded due to the unrelated topic (risk related to exposure to iodine-rich contrast medium during pregnancy). In the final analysis seven paper were included (as illustrated in Fig. 1).

## Relationship between iodine status and pregnancy or neonatal outcomes

In the last eight years, four different meta-analyses have evaluated relationships between iodine status during pregnancy and neonatal and maternal outcomes [23–26] and were selected for the purpose of the present review. Although the topic of these four studies is similar, significant differences both in the evaluated outcomes as well as in the classification of iodine status account for striking differences in the results obtained, as summarized in Table 1.

**Table 1:** Summary of meta-analyzes regarding the relationship between Iodine status and pregnancy or neonatal outcomes

Author	N. of included papers	Maternal/Neonatal outcomes	Evaluation of Iodine status	Results
**Nazeri et al.**[22]	11 on birth weight 7 on birth length 5 on head circumference	growth parameters at birth (birth weight, birth length and head circumference)	Maternal UIC (< 150 vs. > = 150 µg/L)	no significant association between maternal UIC and growth parameters at birth
**Levie et al.**[24]	3 prospective population-based birth cohorts	nonverbal IQ, verbal IQ (evaluated at 1.5 to 8 years of age)	Maternal UI/Creat (deficiency < 150 µg/g, normality 150 to 500 µg/g, excess ≥ 500 µg/g)	positive association of UI/Creat with mean verbal IQ, up to 14 weeks of gestation
**Nazeri et al.**[23]	11 studies for heel blood 10 studies for cord blood	Neonatal TSH (heel or cord sampling)	Maternal UIC during pregnancy (< 150 µg/L for iodine deficiency.) Maternal UIC in early postpartum (< 100 µg/L for iodine deficiency)	No correlation between maternal iodine status and neonatal heel blood TSH Higher neonatal cord blood TSH in iodine deficient mothers (both during pregnancy and post-partum)
**Wan et al.**[25]	5 studies in pregnant women	Maternal TPO Ab and Tg Ab positivity, Maternal TSH	Excess iodine status (UIC > = 500 µg/L) vs. normal (UIC < 500 µg/L)	Higher rates of TgAb and TPO Ab positivity in pregnant women with excess iodine. Higher TSH in pregnant women with excess iodine.

Abbreviations: IQ -Intelligence Quotient; TPO Ab – Anti-Thyroperoxidase Antibodies; Tg Ab- Anti-Thyroglobulin Antibodies; TSH -Thyroid-Stimulating Hormone; UI -Urinary iodine, UIC -Urinary Iodine Concentration, UI/Creat - Urinary iodine/creatinine ratio

WHO definition of Iodine Status: severe iodine deficiency UIC < 20 µg/L, moderate iodine deficiency UIC 20–49 µg/L, mild iodine deficiency UIC 50–99 µg/L, excess iodine UIC > 500 µg/L

First, the four different studies used different thresholds and measurement methods for urinary iodine concentration (UIC) in pregnant women. To define iodine deficiency, the 2016 and the 2020 meta-analyses both by Nazeri et al. [23, 24] evaluated Urinary Iodine Concentration (UIC) during pregnancy using the recommended WHO thresholds of UICs lower than 150 µg/L to define iodine deficiency. The 2016 meta-analysis also included lactating women, using less than 100 µg/L as an UIC threshold. The meta-analysis by Levie et al. [25] included three prospective cohort studies in which maternal iodine status was defined as deficient if the UIC/creatinine was < 150 µg/g, optimal if it was 150 to 500 µg/g, and excessive if it was > 500 µg/g. Finally, the meta-analysis by Wan et al. was limited to studies performed in China and it evaluated only the predictive value of having a UIC above or below 500 µg/L iodine for development of fetal and maternal adverse events [26].

A second important difference between the four studies stems from different outcomes evaluated by each meta-analysis. The 2016 meta-analysis by Nazeri et al., evaluated the associations between pregnancy and post-partum UIC values and several neonatal outcomes, including neonatal TSH as assessed by heel punch and cord blood samples. The 2019 meta-analysis by Levie et al. evaluated offspring neurodevelopment assessed by verbal and non-verbal Intelligence Quotient (IQ) testing at a median 1.5 to 8.6 years of age. The 2020 meta-analysis by Nazeri et al. considered growth parameters at birth, including birth weight, length, and head circumference. Lastly, the study by Wan et al. considered maternal TSH and anti-thyroperoxidase (TPO) and anti-thyroglobulin (Tg) antibody positivity as their main outcome.

These differences clearly impact the conclusions reached by the meta-analyses. The first meta-analysis by Nazeri et al. demonstrated that while there was no correlation between maternal iodine status and neonatal TSH evaluated in heel blood samples, cord blood TSH was significantly higher in newborns of iodine-deficient mothers when compared with children of mothers with adequate iodine levels. The authors concluded that cord blood TSH was a more sensitive parameter for evaluating maternal iodine status when compared with heel blood TSH. The meta-analysis by Levie et al. showed a non-linear correlation between the maternal UI/creatinine ratio and mean verbal IQ of offspring, while no correlation was observed with non-verbal IQ. Stratification of data according to the timing of measurement of UI/creatinine ratio revealed that only values assessed before the 14th gestational week were related to verbal IQ, with no associations when samples were obtained later in pregnancy. These data highlight that the events of the first weeks of pregnancy are essential in determining fetal brain development. These results are particularly relevant since the model employed was adjusted for multiple variables that are known to influence neurodevelopment, including gestational age, child sex, maternal ethnicity/country of birth, maternal education, parity, maternal age, pre-pregnancy body mass index, and smoking during pregnancy. The 2020 meta-analysis by Nazeri et al. failed to show any association between UIC and growth parameters at birth (birth weight, birth length and head circumference). The analysis by Wan et al. showed that excessive iodine levels in pregnant women were associated with a significant increase in the rate of anti-Tg and anti-TPO antibody positivity. Moreover, TSH levels tended to be higher in women with UIC > 500 µg/L as compared to women with lower UIC levels.

## Intervention trials with iodine supplementation in pregnant women

The abundance of observational data regarding the possible detrimental effects of maternal iodine deficiency on neurological development prompted several investigators to design prospective randomized trials to assess the benefits of iodine supplementation during pregnancy. Owing major discrepancies in the results provided by these trials, more recently, several meta-analyses have tried to draw more definitive conclusions (Table 2). It should be noted that although several systematic reviews have been published, in many cases meta-analysis of studies of iodine supplementation in pregnancy was not considered feasible due to heterogeneity of both study selection criteria and lack of controlled trials examining outcomes of interest.

**Table 2:** Summary of meta-analyzes regarding intervention trials with iodine supplementation in pregnant women

Author	Evaluation of iodine supplementation	N. included trials	Maternal/Neonatal Outcomes		Results
**Harding et al.**[27]	injected or oral iodine supplementation during preconception, pregnancy (PR) and postpartum (PP) vs. placebo/no treatment	1 (PR) -3 (PP)	maternal primary outcomes	Hypothyroidism (in pregnancy/post partum)	no difference
		2		Preterm birth	no difference
		1 (PR) -3 (PP)		Elevated TPOAb	no difference
		1 (PR) -3 (PP)		Hyperthyroidism (in pregnancy/post partum)	68% reduction in postpartum hyperthyroidism, no differences in pregnancy
		1		Digestive intolerance	digestive intolerance increased 15 times compared to placebo
		1	infant primary outcomes	Perinatal mortality	trend lower perinatal mortality, not significant
		2		Low birthweight (< 2500 g)	no difference
		1		Neonatal hypothyroidism or elevated TSH	No difference
		1		Neonatal adverse effect: elevated TPOAb	no difference
**Dineva et al.**[28]	Supplementation vs. no supplementation in mildly-to-moderately deficient women (UIC: 50–149 µg/L)	2	maternal thyroid function (TSH; FT4) during pregnancy (second and third trimester)		Lower TSH in second and third trimester in treated women. Lower FT4 only in third trimester in treated women.
		2	Children neurodevelopment (Bayley scores)		no effect of iodine supplementation compared with placebo on child cognitive, language, or motor scores.
**Nazeri et al.**[26]	Any supplementation vs. no supplementation during pregnancy	5	Newborn weight		No difference
		3	Newborn length		No difference
		2	Newborn Head circumference		No difference
		3	Cognitive development		No difference
		3	Language development		No difference
		3	Motor development		No difference

Abbreviations: TPO Ab – Anti-Thyroperoxidase Antibodies; TSH -Thyroid-Stimulating Hormone; UIC -Urinary Iodine Concentration, FT4 - Free-thyroxine

The most comprehensive meta-analysis is represented by the 2017 Cochrane systematic review performed by Harding et al. [28], which was specifically aimed at assessing the benefits and harms of iodine supplementation, alone or in combination with other vitamins and minerals, for women in the preconception, pregnancy or postpartum period on their and their children’s outcomes. The final analysis included eleven trials involving over 2,700 women. Since a high level of heterogeneity among trials was expected by the authors, due to differences in iodine supplement formulations, doses, and duration or timing of interventions, the results were pooled using random-effects models and interpretation was very cautious. The authors considered a wide range of maternal and neonatal outcomes. For the mothers, the main outcomes were maternal hypothyroidism or hyperthyroidism during pregnancy and the post-partum period, preterm birth, elevation of serum TPO antibodies during pregnancy and post-partum, and digestive intolerance for iodine. For newborns, the main outcomes were perinatal mortality, low birthweight, neonatal thyroid dysfunction and neonatal elevation of anti TPO antibodies. The meta-analysis showed that maternal iodine supplementation decreased the likelihood of postpartum hyperthyroidism/thyrotoxicosis by 68%, although these data were derived from three low-quality trials. The only other maternal outcome for which a statistically significant difference was observed was digestive intolerance: pregnant women taking iodine supplementation were 15 times more likely to experience digestive discomfort, although these results came from a single trial in 76 women, again with very low-quality evidence. No significant differences between iodine-supplemented mothers and placebo-treated ones could be found for any of the other outcomes. As for the neonatal outcomes, a non-significant trend towards a 34% reduction in neonatal mortality was observed in children of iodine-supplemented mothers, but it should be noted that all perinatal deaths occurred in a single trial performed in a severely iodine deficient area. For all the other neonatal outcomes, no statistically significant findings were found. The authors concluded that there were insufficient data to reach any meaningful conclusions on the benefits and harms of routine iodine supplementation in women before, during or after pregnancy, due to the low quality of available trials. The authors further specified that it may be unethical to compare iodine to placebo or no treatment in severely iodine deficient settings and that trials may be unfeasible in settings where pregnant and lactating women commonly take iodine-containing prenatal supplements.

More recently, two other meta-analyses attempted to summarize findings of randomized clinical trials of iodine supplementation, with similarly inconclusive results. The meta-analysis by Dineva et al., [29] aimed to evaluate the effects of iodine supplementation in mildly-to-moderately deficient pregnant women on maternal and infant thyroid function and child cognition. Thirty-seven studies were included, although only three were eligible for the meta-analysis. A first meta-analysis included two Randomized-Controlled Trials (RCTs) which administered 200–225 µg Iodine *per* day starting in the first trimester, and assessed maternal TSH and FT4 values in the second and third trimesters [32, 33]. The iodine-supplemented women had lower TSH levels when compared with placebo-treated ones in both the second and the third trimester, while FT4 was significantly lower among supplemented women only in the third trimester. Moreover, median Tg during both the second and the third trimester was significantly lower in the iodine group than in the placebo group. However, the low quality of included studies rendered the results of this meta-analysis non-generalizable.

As for child neurodevelopmental outcomes, the authors were able to perform a meta-analysis on only two RCTs that were comparable in study design, since they both administered iodine as potassium-iodide (KI) tablets in a similar dosage from early pregnancy until delivery, used a placebo as the control treatment, assessed child cognition using the same cognitive tool (Bayley scales), and children were tested at similar ages (1.5 or 2 y) [32, 34]. The analysis showed no effect of iodine supplementation compared with placebo on child cognitive, language, or motor scores. It should be highlighted that one of the two trials considered by the authors was aborted earlier than planned due to funding withdrawal and was substantially underpowered [34]. The meta-analysis concluded that there was insufficient good-quality evidence to support recommendations for iodine supplementation during pregnancy in areas with mild-to-moderate iodine deficiency.

Finally, another very recent meta-analysis by Nazeri et al. [27] aimed to provide comprehensive data on the effects of iodine supplementation in pregnancy, and to investigate potential benefits for infant growth and neurocognitive development. The meta-analytic portion of the paper included five RCTs and evaluated only child outcomes [32, 34–37]. The results of the analysis failed to show any differences in neonatal weight, length, and head circumference at birth, or in cognitive language or motor development during the first 2 years of life in infants between the iodine-supplemented and control groups.

## Discussion

One hundred years have passed since the initiation of iodine supplementation in the United States and in Switzerland [38]. Over the past century, salt iodization has led to the successful eradication of endemic cretinism and severe iodine deficiency in most countries all over the world. It would be expected that univocal evidence should support the role of iodine and iodine supplementation during pregnancy. Meta-analyses are considered among the most valuable tools to inform decision-making in the context of evidence based medicine [39]. The fact that the meta-analyses published so far failed to provide a clear and unifying message, even if much effort was put in the last years in producing evidence on this topic, is rather surprising. Indeed, while some correlation between iodine status and maternal or neonatal outcomes emerges from epidemiological meta-analyses, no effect of iodine supplementation in mildly deficient countries is evidenced by meta-analyses of intervention trials. A direct consequence of this lack of congruence is that surprisingly no clear consensus is available regarding the real advantages of iodine supplementation during pregnancy [40]. Some possible explanation can be envisaged for this lack of straightforward evidence.

The first and most important problem resides in the fact that most studies are currently performed in countries which are only mildly to moderately iodine-deficient. While the damage due to severe iodine deficiency is undoubtable, any neurological consequences of moderate iodine deficiency are likely more subtle and the commonly used outcomes may be insensitive to small differences. The lack of observed effects after iodine supplementation in randomized clinical trials is probably due to the fact that the differences between treated and non-treated subjects are rather small in populations where iodine is widespread (in iodized salt, in processed food and in prenatal multivitamins). On the other hand, performing placebo-controlled clinical trials in countries where severe iodine deficiency is still a reality (where we could expect to observe more evident results) is not considered ethically acceptable [28]. Another factor that could further increase the discrepancies is that almost all of these meta-analyses mix studies conducted in iodine-sufficient areas with studies conducted in mildly and moderately iodine-deficient areas. Only the meta-analysis by Nazeri et al. [23] does not combine studies coming from areas with different iodine status. This mixing could be ​​responsible for a sort of “dilution effect”. In other words, the positive effect of iodine supplementation, which is expected to be more evident in moderately iodine-deficient areas, is diluted by the minimal effect in slightly iodine-deficient areas and by the lack of any effect in iodine-sufficient areas.

As a second point, the available epidemiological studies and clinical trials are extremely variable in terms of study design. First, no standard definition of iodine deficiency during pregnancy is available, and the lack of a common nomenclature makes the comparison across studies difficult, if not impossible. Indeed, while on a population basis a UIC threshold of 150 mcg/L of is conventionally used, this value is not applicable to the individual due to extremely variable within-day and between- day UIC levels, reflecting shifting hydration status and episodic iodine intake [41]. Even when UIC measurement is performed on 24 h urine collections its values are prone to collection and methodological errors [42]. Assessing iodine concentrations in cord blood, placenta or amniotic fluid could lead, at least theoretically, to a more precise assessment of the iodine status of the fetus. Indeed, while UIC is the results of both maternal and fetal iodine status, the direct assessment of iodine in cord blood and amniotic fluid could be more indicative of the status of the fetus only, due to the role of the placenta as a storage organ for iodine[43]. These data could show a more evident correlation between the amount of iodine that is effectively available for the developing fetus and the neurological development outcomes. The few available reports suggest that while cord blood iodine levels are closely related with maternal plasma iodine levels [44], amniotic fluid iodine levels are not correlated with cord blood iodine, with very wide inter-individual variations[45]. Also placental iodine levels have been proposed as a long-term biomarker of gestational iodine load [46]. Unfortunately, these alternatives to UIC are all rather invasive and costly and no validation studies have been performed yet, making them not feasible for iodine status monitoring in pregnancy at least at the moment.

A further aspect stems from the timing of UIC assessment. Indeed, UIC levels vary considerably during pregnancy, with a transient increase in UIC levels in the first trimester due to increased renal iodine clearance and reduced UIC in late pregnancy due to depletion of intra-thyroidal iodine stores, as well as to the iodine shift from the maternal circulation to the growing fetal-placental unit [47]. The timing of UIC sampling can thus profoundly impact data interpretation.

Although some studies try to adjust for variations in glomerular filtration rate using the UIC/creatinine ratio, this is not universally used in epidemiological studies on iodine deficiency [48]. Further inconsistencies derive from the fact that some studies have evaluated only maternal iodine status, while other studies have also evaluated the iodine status of the offspring.

Substantial variability is also present in terms of the outcomes evaluated in the various studies. While some studies have evaluated maternal outcomes (such as thyroid function or obstetric complications), others also consider the neonatal ones (such as growth parameters, neurological alterations and infant thyroid function). In assessing offspring, timing of evaluation has differed widely, since some studies assess outcomes at birth, while other studies include also evaluations later in life. It is worth noting that the meta-analysis by Levie et al. the only one evaluating child outcomes at older ages, demonstrated a non-linear association between maternal urinary iodine and complex child neurological function, i.e. verbal IQ. This finding is particularly relevant since this is the only meta-analysis to date that has adjusted for multiple confounding factors that may impact neurological development.

The timing of outcome evaluation plays an even more relevant role in the intervention trials. Indeed, it is highly probable that the lack of any observed effect in existing trials is in part due to the evaluation of neurological effects too early in the life of the studied children. The consequences of moderate iodine deficiency are best assessed later in life (at least 5 years of age), when more advanced neurological skills can be assessed (such as language and mathematical abilities)[49], but many trials cannot follow patients for so many years. Another issue particularly relevant for intervention trials is the timing of iodine supplementation. Most of the evaluated intervention trials started iodine supplementation at varying times after the beginning of pregnancy, while only two small trials evaluated supplementation when initiated pre-conception. It is biologically plausible that preconception supplementation is more beneficial than starting supplements during gestation, but more specifically designed trials are needed to assess this issue.

Finally, there is likely a relatively narrow window for optimal iodine status in pregnancy, with excess as well as inadequate intakes having the potential to adversely affect maternal thyroid function and child neurological development. Indeed, it is probable that there is a U-shaped association between maternal iodine status and neurological consequences. This U-shaped association was demonstrated by Levie et al. regarding neurological outcomes of offspring. Also the meta-analysis of Wan et al. although limited to the evaluation of the risk related with excess iodine, indirectly supports the need for avoiding over-supplementation. The non-monotonic nature of the relationship between iodine status and clinical outcomes further increases the difficulty in data interpretation, given the lack of a simple linear threshold for defining iodine deficiency.

The reason underlying the detrimental effect of excessive iodine exposure during pregnancy on neurological outcomes could reside in the increase in the incidence of thyroid autoimmunity that is sometimes observed after implementation of iodine-supplementation programs in iodine-deficient countries [50]. Nevertheless, it should be acknowledged that the early post-iodization increase in thyroid antibody positivity is usually transient and not clinically relevant, and the prevalence of overt thyroid dysfunction does not increase in the long-term. If the population median UIC does not exceed the threshold of 300 µg/L, commonly used for indicating iodine-excess, iodine supplementation appears to be safe, with benefits largely outweighing the risk of an increase in thyroid autoimmunity[51].

In conclusion, the four meta-analyses, which have evaluated epidemiological studies on iodine status, suggest that there is a probable correlation between iodine status and several maternal and neonatal outcomes. It is likely that a U-shaped relation exists between UIC and both the risk for maternal thyroid function alterations and children neurological outcomes. Evaluating UIC at the beginning of pregnancy appears to be the most valuable strategy. On the other hand, even if it seems reasonable to state that iodine supplementation during pregnancy might be beneficial, the meta-analyses that took into account the results provided by intervention trials, failed to provide a straightforward conclusion.

However, it is important remembering that there is no controversy about the importance of preventing severe iodine deficiency in pregnancy. Although performed before the advent of modern clinical trial strategies, early supplementation campaigns, as well as studies performed in the 1960 and 1970 s conclusively demonstrated that iodine supplementation in severely deficient regions, can prevent cretinism [52]. The need and modalities of iodine supplementation during pregnancy in moderately or mildly deficient areas remains, instead, still a matter of debate.

To strengthen the evidence supporting iodine supplementation in pregnancy, future trials will need to have the following characteristics i) a randomized-controlled study design ii)collection of precise information on timing, dosing and formulation of iodine supplementation iii) evaluation of the baseline iodine status of the included population, possibly using alternatives to UIC iv) inclusion of information on possible confounders that can impact the offspring neurological outcomes (such as maternal education or exposure to environmental pollutants) v) follow-up information on children neurological development that extends beyond the neonatal period.

In the absence of definitive data coming from meta-analyses, it should be reminded that the current ATA guidelines recommend to start low-dose iodine supplements, optimally 3 months prior to pregnancy in women living in regions of known mild to moderate iodine deficiency [53]. In light of the available evidence, a feasible approach would be to recommend to all pregnant women to regularly consume iodized salt in their diet, adding an oral supplement of 150 µg per day of iodine, in the form of potassium iodide, which should ideally begin at least 3 months before conception. Special care should be given to women at higher risk of iodine deficiency, including women suffering from chronic intestinal malabsorption, coeliac disease or lactose intolerance, or those following specific dietary regimens, such as vegan or low-carbohydrate diets.

## Abbreviations

FT3: Free-Triiodothyronine
FT4: Free-thyroxine
IQ: Intelligence Quotient
KI: Potassium-Iodide
RCT: Randomized-Controlled Trial
Tg: Thyroglobulin
TPO: Thyroperoxidase
TSH: Thyroid-Stimulating Hormone
UI: Urinary iodine
UIC: Urinary Iodine Concentration

## References

[1] Lavado-Autric R, Ausó E, García-Velasco JV, Arufe MeC, Escobar del Rey F, Berbel P, et al. Early maternal hypothyroxinemia alters histogenesis and cerebral cortex cytoarchitecture of the progeny. J Clin Invest. 2003;111(7):1073-82. 10.1172/JCI16262. 10.1172/JCI16262 PMC152582 12671057

[2] Zimmermann MB (2011). The role of iodine in human growth and development. Semin Cell Dev Biol.

[3] Leung AM, Pearce EN, Braverman LE (2011). Iodine nutrition in pregnancy and lactation. Endocrinol Metab Clin North Am.

[4] Bath SC, Steer CD, Golding J, Emmett P, Rayman MP (2013). Effect of inadequate iodine status in UK pregnant women on cognitive outcomes in their children: results from the Avon Longitudinal Study of Parents and Children (ALSPAC). Lancet.

[5] Zimmermann MB, Jooste PL, Pandav CS (2008). Iodine-deficiency disorders. Lancet.

[6] Rotondi M, Amato G, Biondi B, Mazziotti G, Del Buono A, Rotonda Nicchio M (2000). Parity as a thyroid size-determining factor in areas with moderate iodine deficiency. J Clin Endocrinol Metab.

[7] Andersson M, Aeberli I, Wüst N, Piacenza AM, Bucher T, Henschen I (2010). The Swiss iodized salt program provides adequate iodine for school children and pregnant women, but weaning infants not receiving iodine-containing complementary foods as well as their mothers are iodine deficient. J Clin Endocrinol Metab.

[8] Wong EM, Sullivan KM, Perrine CG, Rogers LM, Peña-Rosas JP (2011). Comparison of median urinary iodine concentration as an indicator of iodine status among pregnant women, school-age children, and nonpregnant women. Food Nutr Bull.

[9] World Health Organization. Assessment of iodine deficiency disorders and monitoring their elimination: a guide for programme managers. Available at http://apps.who.int/iris/bitstream/handle/10665/43781/9789241595827_eng.pdf (2007).

[10] Chittimoju SB, Pearce EN (2019). Iodine Deficiency and Supplementation in Pregnancy. Clin Obstet Gynecol.

[11] Hynes KL, Otahal P, Hay I, Burgess JR (2013). Mild iodine deficiency during pregnancy is associated with reduced educational outcomes in the offspring: 9-year follow-up of the gestational iodine cohort. J Clin Endocrinol Metab.

[12] van Mil NH, Tiemeier H, Bongers-Schokking JJ, Ghassabian A, Hofman A, Hooijkaas H (2012). Low urinary iodine excretion during early pregnancy is associated with alterations in executive functioning in children. J Nutr.

[13] Abel MH, Caspersen IH, Meltzer HM, Haugen M, Brandlistuen RE, Aase H (2017). Suboptimal Maternal Iodine Intake Is Associated with Impaired Child Neurodevelopment at 3 Years of Age in the Norwegian Mother and Child Cohort Study. J Nutr.

[14] Murcia M, Espada M, Julvez J, Llop S, Lopez-Espinosa MJ, Vioque J (2018). Iodine intake from supplements and diet during pregnancy and child cognitive and motor development: the INMA Mother and Child Cohort Study. J Epidemiol Community Health.

[15] Abel MH, Ystrom E, Caspersen IH, Meltzer HM, Aase H, Torheim LE, et al. Maternal Iodine Intake and Offspring Attention-Deficit/Hyperactivity Disorder: Results from a Large Prospective Cohort Study. Nutrients. 2017;9(11). 10.3390/nu9111239. 10.3390/nu9111239 PMC5707711 29137191

[16] Hynes KL, Otahal P, Burgess JR, Oddy WH, Hay I. Reduced Educational Outcomes Persist into Adolescence Following Mild Iodine Deficiency in Utero, Despite Adequacy in Childhood: 15-Year Follow-Up of the Gestational Iodine Cohort Investigating Auditory Processing Speed and Working Memory. Nutrients. 2017;9(12). 10.3390/nu9121354. 10.3390/nu9121354 PMC5748804 29236073

[17] Ghassabian A, Steenweg-de Graaff J, Peeters RP, Ross HA, Jaddoe VW, Hofman A (2014). Maternal urinary iodine concentration in pregnancy and children’s cognition: results from a population-based birth cohort in an iodine-sufficient area. BMJ Open.

[18] Murcia M, Rebagliato M, Iñiguez C, Lopez-Espinosa MJ, Estarlich M, Plaza B (2011). Effect of iodine supplementation during pregnancy on infant neurodevelopment at 1 year of age. Am J Epidemiol.

[19] Taylor PN, Vaidya B (2016). Iodine supplementation in pregnancy - is it time?. Clin Endocrinol (Oxf).

[20] Shi X, Han C, Li C, Mao J, Wang W, Xie X (2015). Optimal and safe upper limits of iodine intake for early pregnancy in iodine-sufficient regions: a cross-sectional study of 7190 pregnant women in China. J Clin Endocrinol Metab.

[21] Sang Z, Wei W, Zhao N, Zhang G, Chen W, Liu H (2012). Thyroid dysfunction during late gestation is associated with excessive iodine intake in pregnant women. J Clin Endocrinol Metab.

[22] Medici M, Ghassabian A, Visser W, de Keizer-Schrama M, Jaddoe SM, Visser VW (2014). Women with high early pregnancy urinary iodine levels have an increased risk of hyperthyroid newborns: the population-based Generation R Study. Clin Endocrinol (Oxf).

[23] Nazeri P, Shab-Bidar S, Pearce EN, Shariat M (2020). Do maternal urinary iodine concentration or thyroid hormones within the normal range during pregnancy affect growth parameters at birth? A systematic review and meta-analysis. Nutr Rev.

[24] Nazeri P, Mirmiran P, Kabir A, Azizi F (2016). Neonatal thyrotropin concentration and iodine nutrition status of mothers: a systematic review and meta-analysis. Am J Clin Nutr.

[25] Levie D, Korevaar TIM, Bath SC, Murcia M, Dineva M, Llop S (2019). Association of Maternal Iodine Status With Child IQ: A Meta-Analysis of Individual Participant Data. J Clin Endocrinol Metab.

[26] Wan S, Jin B, Ren B, Qu M, Wu H, Liu L (2020). The Relationship between High Iodine Consumption and Levels of Autoimmune Thyroiditis-Related Biomarkers in a Chinese Population: a Meta-Analysis. Biol Trace Elem Res.

[27] Nazeri P, Shariat M, Azizi F (2021). Effects of iodine supplementation during pregnancy on pregnant women and their offspring: a systematic review and meta-analysis of trials over the past 3 decades. Eur J Endocrinol.

[28] Harding KB, Peña-Rosas JP, Webster AC, Yap CM, Payne BA, Ota E (2017). Iodine supplementation for women during the preconception, pregnancy and postpartum period. Cochrane Database Syst Rev.

[29] Dineva M, Fishpool H, Rayman MP, Mendis J, Bath SC (2020). Systematic review and meta-analysis of the effects of iodine supplementation on thyroid function and child neurodevelopment in mildly-to-moderately iodine-deficient pregnant women. Am J Clin Nutr.

[30] van Welie N, Portela M, Dreyer K, Schoonmade LJ, van Wely M, Mol BWJ (2021). Iodine contrast prior to or during pregnancy and neonatal thyroid function: a systematic review. Eur J Endocrinol.

[31] Taylor PN, Okosieme OE, Dayan CM, Lazarus JH (2014). Therapy of endocrine disease: Impact of iodine supplementation in mild-to-moderate iodine deficiency: systematic review and meta-analysis. Eur J Endocrinol.

[32] Gowachirapant S, Jaiswal N, Melse-Boonstra A, Galetti V, Stinca S, Mackenzie I (2017). Effect of iodine supplementation in pregnant women on child neurodevelopment: a randomised, double-blind, placebo-controlled trial. Lancet Diabetes Endocrinol.

[33] Censi S, Watutantrige-Fernando S, Groccia G, Manso J, Plebani M, Faggian D, et al. The Effects of Iodine Supplementation in Pregnancy on Iodine Status, Thyroglobulin Levels and Thyroid Function Parameters: Results from a Randomized Controlled Clinical Trial in a Mild-to-Moderate Iodine Deficiency Area. Nutrients. 2019;11(11). 10.3390/nu11112639. 10.3390/nu11112639 PMC6893432 31689890

[34] Zhou SJ, Skeaff SA, Ryan P, Doyle LW, Anderson PJ, Kornman L (2015). The effect of iodine supplementation in pregnancy on early childhood neurodevelopment and clinical outcomes: results of an aborted randomised placebo-controlled trial. Trials.

[35] Velasco I, Carreira M, Santiago P, Muela JA, García-Fuentes E, Sánchez-Muñoz B (2009). Effect of iodine prophylaxis during pregnancy on neurocognitive development of children during the first two years of life. J Clin Endocrinol Metab.

[36] Brucker-Davis F, Ganier-Chauliac F, Gal J, Panaïa-Ferrari P, Pacini P, Fénichel P (2015). Neurotoxicant exposure during pregnancy is a confounder for assessment of iodine supplementation on neurodevelopment outcome. Neurotoxicol Teratol.

[37] Hiéronimus S, Ferrari P, Gal J, Berthier F, Azoulay S, Bongain A (2013). Relative impact of iodine supplementation and maternal smoking on cord blood thyroglobulin in pregnant women with normal thyroid function. Eur Thyroid J.

[38] Leung AM, Braverman LE, Pearce EN (2012). History of U.S. iodine fortification and supplementation. Nutrients.

[39] Berlin JA, Golub RM (2014). Meta-analysis as evidence: building a better pyramid. JAMA.

[40] Andersen SL, Laurberg P (2016). Iodine Supplementation in Pregnancy and the Dilemma of Ambiguous Recommendations. Eur Thyroid J.

[41] Hlucny K, Alexander BM, Gerow K, Larson-Meyer DE. Reflection of Dietary Iodine in the 24 h Urinary Iodine Concentration, Serum Iodine and Thyroglobulin as Biomarkers of Iodine Status: A Pilot Study. Nutrients. 2021;13(8). 10.3390/nu13082520. 10.3390/nu13082520 PMC8398459 34444680

[42] Remer T, Fonteyn N, Alexy U, Berkemeyer S (2006). Longitudinal examination of 24-h urinary iodine excretion in schoolchildren as a sensitive, hydration status-independent research tool for studying iodine status. Am J Clin Nutr.

[43] Burns R, O’Herlihy C, Smyth PP (2011). The placenta as a compensatory iodine storage organ. Thyroid.

[44] Rayburn WF, Robinson A, Braverman LE, He XM, Pino S, Gargas ML (2008). Iodide concentrations in matched maternal serum, cord serum, and amniotic fluid from preterm and term human pregnancies. Reprod Toxicol.

[45] Zou Y, Wang D, Yu S, Cheng X, Xia L, Yin Y (2020). Rapid inductively coupled plasma mass spectrometry method to determine iodine in amniotic fluid, breast milk and cerebrospinal fluid. Clin Biochem.

[46] Neven KY, Marien CBD, Janssen BG, Roels HA, Waegeneers N, Nawrot TS (2020). Variability of iodine concentrations in the human placenta. Sci Rep.

[47] Brander L, Als C, Buess H, Haldimann F, Harder M, Hänggi W (2003). Urinary iodine concentration during pregnancy in an area of unstable dietary iodine intake in Switzerland. J Endocrinol Invest.

[48] Luo J, Li C, Zhang X, Shan Z, Teng W (2021). Reference Intervals of the Ratio of Urine Iodine to Creatinine in Pregnant Women in an Iodine-Replete Area of China. Biol Trace Elem Res.

[49] Bargagna S, Canepa G, Costagli C, Dinetti D, Marcheschi M, Millepiedi S (2000). Neuropsychological follow-up in early-treated congenital hypothyroidism: a problem-oriented approach. Thyroid.

[50] Locantore P, Corsello A, Policola C, Pontecorvi A (2021). Subclinical thyroid diseases and isolated hypothyroxinemia during pregnancy. Minerva Endocrinol (Torino).

[51] Ruggeri RM, Trimarchi F (2021). Iodine nutrition optimization: are there risks for thyroid autoimmunity?. J Endocrinol Invest.

[52] Pharoah PO. Iodine-supplementation trials. Am J Clin Nutr. 1993;57(2 Suppl):276S-9S. 10.1093/ajcn/57.2.276S. 10.1093/ajcn/57.2.276S 8427204

[53] Alexander EK, Pearce EN, Brent GA, Brown RS, Chen H, Dosiou C (2017). 2017 Guidelines of the American Thyroid Association for the Diagnosis and Management of Thyroid Disease During Pregnancy and the Postpartum. Thyroid.

